# Aging, not Parkinson’s disease, decreases a recalibration of body ownership caused by vision-respiratory interaction

**DOI:** 10.3389/fphys.2024.1419473

**Published:** 2024-10-31

**Authors:** Daiki Shoji, Motoyasu Honma, Yuri Masaoka, Momoka Nakayama, Miku Kosuge, Shota Kosuge, Yuki Uchida, Shunsuke Sakakura, Misako Matsui, Naohito Ito, Tetsuhito Nohara, Daishi Watanabe, Mizuki Kanemoto, Hideyo Kasai, Takeshi Kuroda, Satoshi Yano, Hidetomo Murakami, Masahiko Izumizaki

**Affiliations:** ^1^ Department of Physiology, Showa University School of Medicine, Tokyo, Japan; ^2^ Department of Neurology, Showa University School of Medicine, Tokyo, Japan; ^3^ Dentsu Lab Tokyo, Dentsu Inc., Tokyo, Japan; ^4^ Department of Respiratory Medicine, Showa University Fujigaoka Hospital, Yokohama, Japan; ^5^ Department of Neurology, Showa University Northern Yokohama Hospital, Yokohama, Japan

**Keywords:** body ownership, rubber hand illusion, respiratory rhythm, aging, Parkinson’s disease

## Abstract

**Introduction:**

Recalibration of body ownership perception occurs through an integration among multiple modalities. A recent study has shown that respiratory rhythm also causes the recalibration of ownership perception. However, the risk factors influencing the recalibration of ownership perception caused by vision-respiratory interaction remain unclear. In this study, focusing on aging and Parkinson’s disease (PD), we examined the effects of those risk factors on the recalibration.

**Methods:**

By applying the rubber hand illusion (RHI), which temporarily alters ownership perception, and using a device that synchronizes the respiratory rhythm with the movement of a mannequin hand, we measured a change in ownership perception in RHI training by vision-respiratory interaction. The changed ownership was compared among the elderly healthy, PD, and young healthy groups.

**Results:**

The results showed no difference in the changed ownership between the elderly healthy and PD groups, while the two groups decreased the change in the ownership perception compared to the young healthy group.

**Discussion:**

The finding suggests that aging, not PD, related to the recalibration of ownership perception by vision-respiratory interaction. An anomaly in body perception due to aging may be associated with a mechanism in which respiratory rhythm affects the adaptation of body representations.

## 1 Introduction

Through everyday experience, internal models of the brain temporally and spatially constrain the interpretation of modality input in forming coherent perceptions of body ownership (Tsakiris, 2010; Blanke, 2012). On shorter time scales, neural representations of the body strategically adapt to changes in behavioral demands, such as during tool use (Maravita and Iriki, 2004). In the classic rubber hand illusion (RHI), when an artificial hand and participant’s hand are synchronously touched by the tactile stimuli in a state where they keep looking at the artificial hand, the participant is perceived as if the artificial hand were part of one’s own body (Botvinick and Cohen, 1998). The RHI causes a recalibration of the spatial location of visual and somatosensory modalities (including tactile and proprioception), which distorts the observer’s perception of the hand’s spatial location and instills an ownership in the artificial hand (Longo et al., 2008). Multimodality recalibration is a common process that updates the relationships between visual, auditory, and somatosensory signals and adjusts for spatial or temporal offsets between the modalities (Chen and Vroomen, 2013; Recanzone, 1998; Van der Burg et al., 2013).

Body ownership perception is established by combining a variety of modality information such as vision, hearing, and somatesthesia (touch and proprioception). However, are modalities the only information needed to recalibrate body ownership perception? One candidate information that might contribute to the recalibration of body perception is respiration. The most important function of respiration is gas exchange, while it is also known to affect various cognitive functions. For example, it has been reported that manipulating the depth of respiration improves memory and cognitive abilities (Vlemincx et al., 2009; Heck et al., 2019; Zelano et al., 2016). On the other hand, the depth and rhythm of respiration can be altered by various factors, such as stress (Grassmann et al., 2016; Kuroda et al., 2012). Thus, respiration and cognition influence each other, and a physiological interrelationship has been proposed in terms of the relationship between neuronal oscillations of respiration and brain function (Maric et al., 2020; Varga and Heck, 2017). A recent study demonstrated the phenomenon of altered ownership perception in RHI in healthy individuals when observing a mannequin hand moving in real-time conjunction with respiratory rhythms (Kosuge et al., 2023). This finding suggests that respiratory rhythm is involved in the recalibration of ownership and that the temporal and spatial congruence of respiration rhythm and visual information is important. However, much is unclear regarding the recalibration of ownership perception caused by vision-respiratory interaction.

In this study, we took a neuropsychological approach to explore the risk factors for the recalibration of ownership perception by vision-respiratory interaction. The factors we focused on were aging and Parkinson’s disease (PD). Although there are many reports that aging decreases various functions (Park and Reuter-Lorenz, 2009; Jessen et al., 2020), it has been reported that in integrated functions, aging is unlikely to cause a decrease in integrated functions such as vision, touch, or hearing (Riemer et al., 2019; DeLoss et al., 2013). Furthermore, it has been reported that age factors do not play a role in the generation of RHI (Campos et al., 2021). We investigated whether age-related differences in the RHI might be a factor in the decline of recalibration of ownership caused by vision and respiratory rhythm. Based on previous studies of integrated function, it is expected that the effects of aging are minimal. On the other hand, PD has been reported to decrease various cognitive functions as well as motor impairment (Reddy et al., 2002; Cools, 2006; Goldman and Weintraub, 2015; Mahlknecht et al., 2016; Huang et al., 2023), and has also been shown to reduce the integrated function of vision and olfactory (Honma et al., 2018a). Based on that previous study, it is likely that PD impairs the recalibration of ownership caused by vision and respiratory rhythm.

In this study, to investigate one aspect of the mechanism of ownership perception, we focused on the latest RHI, which occurs through the interaction of vision and respiration, and examined the recalibration of ownership perception that occurs through RHI training. We measured the two indexes of subjective evaluation of ownership perception and location perception of own’s hand in the RHI paradigm, and the differences before/after RHI training were defined as the amount of the recalibration. To investigate the effect of aging and PD on recalibration of body ownership caused by vision and respiratory rhythm, we compared the healthy young group, the healthy elderly group, and the PD group.

## 2 Materials and methods

### 2.1 Participants

This study was approved by the Ethics Committee of the Showa University School of Medicine and was conducted in accordance with the principles of the Helsinki Declaration (study identification number: 22–157-B). Thirty-two healthy elderly participants (no history of neurological or psychiatric disorders) and 32 PD patients provided written informed consent before the study (Table 1). Participants were paid ¥3,000 as a gratuity. Data for 38 young healthy participants was used from Kosuge et al. (2023) which had the same device and design. All participants had normal vision with or without correction. SPA-5 (Five-item Subjective and Personal Agency scale) and STAI (State-Trait Anxiety Inventory) assessments were conducted to determine psychiatric illness characteristics of all participants. In addition, the elderly and PD participants were assessed with the MMSE (Mini Mental State Examination) for cognitive decline and the UPDRS-III (Unified Parkinson’s Disease Rating Scale, Part III) for Parkinson’s disease severity.

**TABLE 1:** Participants details.

	YH	EH	PD
Number of participants	38	32	32
Numbers of women	17	14	15
Age	22.2 (1.7)	72.5 (4.9)	71.0 (11.0)
SPA-5	16.6 (3.8)	21.3 (3.3)	18.6 (4.1)
STAI-state	35.7 (9.4)	41.1 (6.6)	36.5 (8.6)
STAI-trait	41.5 (10.9)	36.4 (6.7)	41.9 (9.7)
MMSE	-	29.3 (0.9)	28.8 (1.7)
UPDRS-III	-	2.6 (2.4)	23.8 (12.1)

YH: Young health group, EH: Elderly health group, PD: Parkinson’s disease group, SPA-5: Five-item Subjective and Personal Agency scale, STAI: State-Trait Anxiety Inventory, MMSE: Mini-Mental State Examination, UPDRS-III: Unified Parkinson’s disease rating scale-Part III., the standard deviations are shown in parentheses.

### 2.2 Apparatus

**FIGURE 1:**
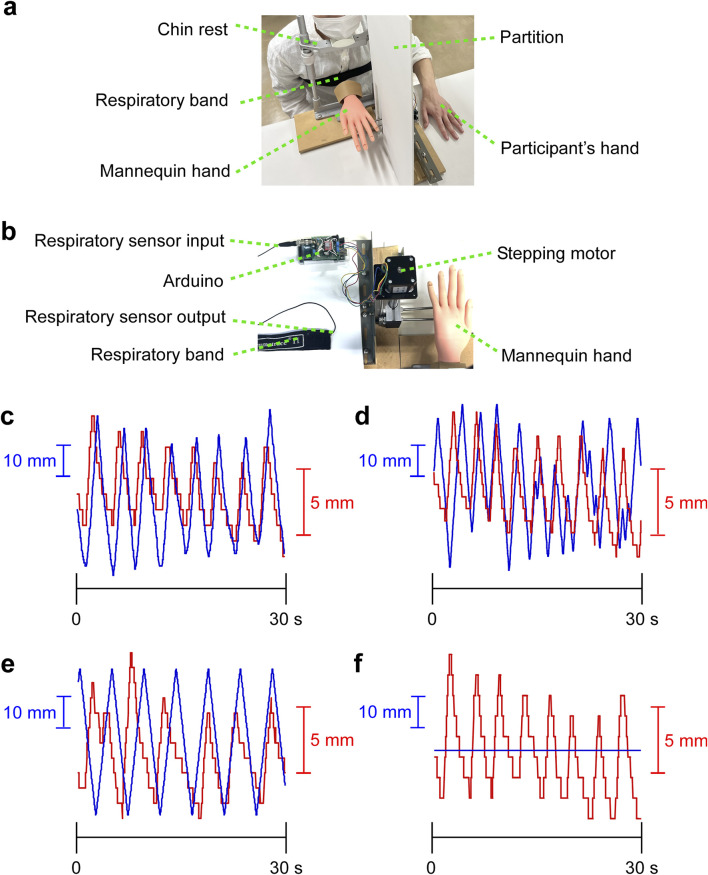
Experimental procedure. **(A)** Experimental environment. The mannequin hand moved up and down in real-time in conjunction with the respiratory rhythm. **(B)** Sensor and device. A respiratory band is wrapped around the participant’s chest, and waveform data of the respiratory rhythm is acquired from the expansion and contraction of the chest. The waveform data is reflected in real-time in the vertical movement of the mannequin hand. Analog data from the respiration sensor was input to the Arduino, and the respiration data was converted to digital data from 0 to 255. Based on the respiration data, the stepping motor was controlled from a PC via the Arduino. **(C–F)** Representative movements of the mannequin hand and respiratory band. The graphs with 30 s extracted from a 2-min trial. Blue lines indicate vertical movements of mannequin hand and red lines indicate elastic movement of respiratory band. **(C)** Temporal synchronization condition (cross-correlation coefficient: r = 0.795). **(D)** Temporal asynchronization condition (r = 0.269). **(E)** Constant speed condition (r = 0.01). **(F)** Static condition.

A device was created in which the mannequin’s hand moved up and down in response to expansion and contraction of respiratory band (Figure 1A). Respiratory rhythm was measured using a breast band (Respitrace system, AMI, Ardsley, NY) (Izumizaki et al., 2008). The control system was created in the programming language Processing, which acquires waveform signals from the expansion and contraction of the respiratory band and reflects them in the vertical movement of the mannequin in real-time (average delay 0.11 s) (Figure 1B). The maximum range of motion of the mannequin was 10 cm (approximately 2 cm of movement of the respiratory band corresponds to 8 cm of movement of the mannequin). The starting point of the mannequin was 6 cm above the desk (in the center of the range of motion). Each participant was seated in a chair, chin resting on a chin rest, head secured with a headband to prevent body movement. The mannequin’s left hand was positioned in front of the participant, and the participant’s left middle finger was positioned 25 cm to the left of the mannequin’s left middle finger. The participant’s left hand was not directly observable by partition. To minimize the influence of environmental noise during the experiment, participants were asked to wear headphones, and white noise was played. The start of the white noise was used as a cue to start focusing their gaze on the mannequin hand.

### 2.3 Experimental condition

Three factors were established for this experiment: spatial, temporal, and group factors. Four conditions of temporal factor were established for a relationship between the participant’s respiratory rhythm and the vertical movement of the mannequin’s hand. For the synchronization condition, the mannequin’s vertical movement was synchronized with the participant’s respiratory rhythm (Figure 1C; Supplementary Videos S1, S5); for the asynchronization condition, the mannequin’s movement was random and smooth using a purlin noise function (Figure 1D; Supplementary Videos S2, S6); for the constant speed condition, the mannequin’s movement was in a constant rhythm (Figure 1E; Supplementary Videos S3, S7); and for the static condition, the mannequin’s movement was stationary (Figure 1F; Supplementary Videos S4, S8). For the spatial factor, the congruency condition was defined as when the mannequin and the participant’s fingers pointed in the same direction, and the incongruency condition was defined as when the mannequin’s fingertips were pointing toward the participant and the palm was pointing upward. A total of eight conditions were performed in random order for each participant. In addition, a cross-correlation coefficient was calculated to examine the degree of synchronization between respiratory band and mannequin hand. We filmed a video from a perspective that showed both the device and the participants, and analyzed the synchronization between the left-right movement of the respiratory band and the up-down movement of the mannequin hand in the video.

### 2.4 Procedure

Participants were instructed to observe the mannequin during RHI training (2 min). They conducted an evaluation of location perception and a subjective evaluation of ownership perception before and after the training (Honma et al., 2016; Honma et al., 2018b). In the measurement of location perception, participants were asked to estimate the specific position of the middle finger of the left hand, and to draw a straight line using the right hand on a white paper attached to the back of a desk. The center point of the drawn line was used as the location perception point, and the distance from the left edge of the desk was calculated. In the measurement of subjective evaluation of ownership perception, participants were asked to draw a vertical line (visual analog scale: VAS) on a straight line 100 mm wide to indicate “how much the mannequin hand felt like their own hand” (extreme left side: not at all, extreme right side: strongly feel). The analysis was performed in millimeters and measured the distance from the extreme left. The range of the VAS was from 0 to 100. For example, if a participant strongly feels the rubber hand as your own hand, the participant would place a check mark 90 mm from the leftmost point. In this case, the VAS score would be 90. The amount of changed ownership and location perception were calculated as the difference between before and after the RHI training. Participants took a 2-minute break between conditions, and their heads were immobilized during the break.

### 2.5 Statistical analysis

The numerical values for each changed ownership perception and changed location perception were calculated as the difference between before and after the RHI training. A three-way ANOVA was performed to test the main effects and interaction of temporal factor (synchronization, asynchronization, constant, and static), spatial factor (congruent and incongruent), and group factor (young, elderly, and PD). Post-hoc t-tests with Bonferroni correction were performed to test multiple comparisons. All tests were two-tailed. The results are presented as mean ± standard error of the mean, effect sizes (*η* ^2^). SPSS 26.0 (IBM Corp., Armonk, NY) was used for statistical analysis.

## 3 Results

An elderly participant and 2 patients with Parkison’s disease were removed from the analysis because they withdrew during the study. Overall, there were group differences in the ownership, while not in the location sense. Furthermore, in the temporal synchronization and spatial congruence condition, the elderly and PD groups decreased the changed ownership perception than the young group, and there was no difference between the elderly and PD groups in the changed ownership.

**FIGURE 2:**
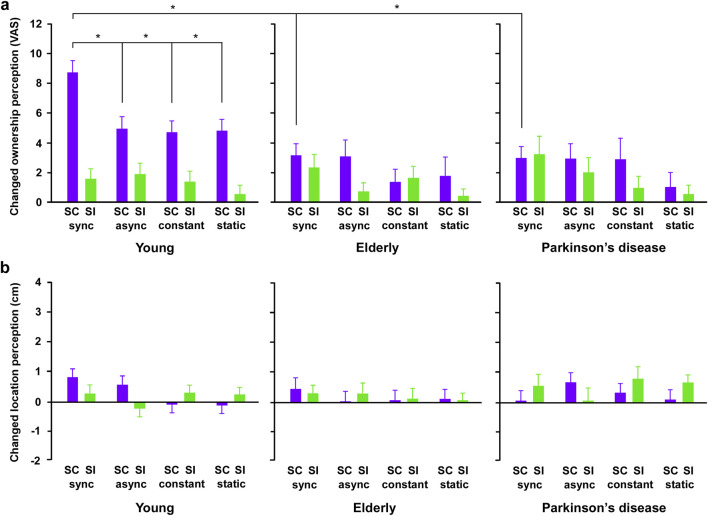
The results of ownership and location perception in all groups. **(A)** The results of changed ownership perception. In the spatial congruency conditions, changed ownership sense increased compared to other conditions in young, not elderly and Parkinson’s disease groups. In the combination condition of the synchronization and spatial congruence, the changed ownership in the healthy young group increased compared to that in the elderly and PD group, while there was no difference in the changed ownership between the elderly and PD groups. **(B)** The results of changed location perception. There was no difference in the changed location sense between spatial congruency (SC) and spatial incongruency (SI) conditions in all groups. There was no difference in the changed location sense among the four temporal conditions in all groups. Error bars show the standard error of the mean. VAS: visual analog scale, SC: spatial congruency condition, SI: spatial incongruency condition, sync: synchronization condition, async: asynchronization condition, constant: constant speed condition, static: static condition. Asterisks mean a significant difference (*p* < 0.05).

In the ownership perception (Figure 2A), three-way ANOVA showed that there were significant differences in the main effect of spatial factor (congruent and incongruent) (F_3, 288_ = 6.128, *p* < 0.0001, *η* ^2^ = 0.060) and temporal factors (synchronization, asynchronization, constant, and static) (F_1, 96_ = 21.355, *p* < 0.0001, *η* ^2^ = 0.182), and no main effect of group (young, elderly, and PD) (F_1, 96_ = 2.010, *p* = 0.140, *η* ^2^ = 0.040). The interaction of group and spatial factors was significantly different (F_2, 96_ = 5.168, *p* = 0.007, *η* ^2^ = 0.097), while there was no main effect of interaction between group and temporal factors (F_6, 288_ = 0.367, *p* = 0.899, *η* ^2^ = 0.008) and between spatial and temporal factors (F_3, 288_ = 0.328, *p* = 0.805, *η* ^2^ = 0.003). The interaction among group, temporal, and spatial factors was a significant difference (F_6, 288_ = 2.372, *p* = 0.030, *η* ^2^ = 0.047). Post-hoc tests revealed that, in the young group, the changed ownership sense in the synchronization condition increased compared to that in other temporal conditions under the spatial congruent conditions (all *p* < 0.05). Furthermore, in all temporal conditions, the changed ownership in the spatial congruent condition increased compared to that in the incongruent condition (all *p* < 0.05). In contrast, in the elderly and PD groups, three-way ANOVA showed that there was no significant difference in all main effects and interactions. Note, in the combination condition of the temporal synchronization and spatial congruence, the changed ownership sense in the young group increased compared to that in the elderly and PD groups (respectively *p* < 0.05), while there was no difference in the changed ownership sense between the elderly and PD groups.

In the location perception (Figure 2B), three-way ANOVA showed that there were no differences in the main effect of spatial (F_1, 96_ = 0.214, *p* = 0.645, *η* ^2^ = 0.002) and temporal (F_3, 288_ = 0.491, *p* = 0.689, *η* ^2^ = 0.005), and group (F_2, 96_ = 0.742, *p* = 0.479, *η* ^2^ = 0.016). No main effects were the interaction between spatial with group factors (F_2, 96_ = 0.417, *p* = 0.660, *η* ^2^ = 0.009), temporal with group factors (F_6, 288_ = 0.398, *p* = 0.880, *η* ^2^ = 0.008), spatial with temporal factors (F_3, 288_ = 1.313, *p* = 0.271, *η* ^2^ = 0.014), and among spatial, temporal, and group factors (F_6, 288_ = 0.948, *p* = 0.461, *η* ^2^ = 0.020).

## 4 Discussion

Unlike the young group, the changed ownership was few in the elderly and PD groups. Furthermore, the changed ownership in the combined spatial congruence and temporal synchronization condition was decreased in the elderly and PD groups than in the young group. There was also no difference between the elderly and PD groups in all conditions. These results suggest that aging, but not PD, factors are involved in the recalibration of body ownership caused by vision-respiratory interaction.

There are two possible reasons for the results. One is a decline in the integration function and the other is a decline in the attention function. While aging degrades various functions, previous studies have shown that the integrated function between the various modalities is less affected by aging (Riemer et al., 2019; DeLoss et al., 2013). It has been suggested that the integrated function of multiple modal cues may enhance to compensate for individual modality ability deteriorated by aging (Chancel et al., 2018). However, the current study observed that the recalibration of ownership caused by vision and respiratory rhythm is affected by aging. This suggests that aging has exacerbated, or not compensated for, the recalibration of ownership caused by vision-respiration interaction. The integration between vision and respiration may be a different mechanism than integration between modalities. Integration between modalities is associated with the insula cortex and median and parietal regions (Bushara et al., 2001; Kuroki et al., 2018). On the other hand, the association between cognition and respiratory may involve the olfactory bulb, piriform cortex, amygdala, and orbitofrontal cortex (Zeller et al., 2011; Ito et al., 2014; Grosmaitre et al., 2007; Linster and Cleland, 2016; Soudry et al., 2011). This difference in brain processing may have influenced the effects of aging factors.

Another possibility is that the spatial attention function may have declined due to aging. Previous studies have shown a decrease with age in various attention functions including spatial attention (Verhaeghen and Cerella, 2002; Vallesi et al., 2021). The prefrontal cortex and posterior parietal cortex are critical neural substrates for persistent activity during the maintenance of spatial attention (Ikkai and Curtis, 2011), and functions of the regions decline with age (Jobson et al., 2021). Suppose we apply this to the current results, it is possible that spatial attention maintenance for rubber hand has declined, and that this caused the decline in recalibration of ownership caused by vision-respiratory interaction. These two possibilities need to be examined in the future.

PD has been shown to deplete dopamine neurons in the substantia nigra and the striatum, resulting in a variety of impaired brain functions. It is mainly characterized by motor impairments such as tremor and postural retention deficits (Greenbaum et al., 2013), however, it also causes various cognitive dysfunctions other than motor impairment, including attention, working memory, response inhibition, and task switching (Manza et al., 2017; Ramos and Machado, 2021; Cavanagh et al., 2022). Although there are few studies of integrated function in PD, one study reported a decline in the integrated function of visual and olfactory (Honma et al., 2018a). In the present experiment, however, there was little difference in results between PD and elderly persons, suggesting that PD has little effect on the attention function and/or integrated function of vision and respiratory rhythm. In other words, the basal ganglia, including the striatum, are likely less involved in the recalibration of ownership perception caused by vision-respiration interaction, highlighting the strong influence of other regions that are diminished by aging.

The study has several limitations. First, the tidal volume of respiration was not measured in this experiment. This was because a mask-type respiratory measurement device could not be used during the COVID-19 epidemic. In the future, the influence of individual respiratory volume on the recalibration of ownership perception should be investigated. Second, this study relied on subjective reports of ownership and drift in location perception by the VAS. With this method, it is difficult to determine whether differences associated with age are specific to changes in ownership perception or due to changes in an internal criterion to report ownership perception. Using a signal detection theory (discrimination task in RHI) (Lanfranco et al., 2023; Lanfranco et al., 2024), it is possible to identify a decision criterion, and may distinguish between the aspects of sensitivity and decision criterion for ownership in age-related differences. Third, concerning the brain mechanism, this study only denied the influence of the basal ganglia by comparing young healthy subjects, elderly healthy subjects, and PD patients. Future studies should use fMRI and other techniques to identify brain regions that play a central role in the integrated function of vision and respiration.

In conclusion, the present study used a neuropsychological approach to examine the effects of integration of vision and respiratory rhythm on changes in body perception, suggesting a strong involvement of aging factors rather than PD. Although we are rarely aware of the link between respiration and body perception in everyday life, respiration may be constantly involved in the readjustment of body perception. An anomaly in body perception due to aging may be associated with a mechanism in which respiratory rhythm affects the adaptation of body representations.

## Data Availability

The raw data supporting the conclusions of this article will be made available by the authors, without undue reservation.

## Supplementary material

SUPPLEMENTARY VIDEO S1 Representative video in the conditions of spatial congruence and temporal synchronization. The mannequin and the participant’s fingers pointed in the same direction. The left-right movement of the respiratory band and the up-down movement of the mannequin hand were synchronized.

SUPPLEMENTARY VIDEO S2 Representative video in the conditions of spatial congruence and temporal asynchronization. The mannequin and the participant’s fingers pointed in the same direction. The left-right movement of the respiratory band and the up-down movement of the mannequin hand were asynchronous due to random movement of mannequin hand.

SUPPLEMENTARY VIDEO S3 Representative video in the conditions of spatial congruence and temporal constant speed. The mannequin and the participant’s fingers pointed in the same direction. The left-right movement of the respiratory band and the up-down movement of the mannequin hand were asynchronous due to constant movement of the mannequin hand.

SUPPLEMENTARY VIDEO S4 Representative video in the conditions of spatial congruence and temporal static. The mannequin and the participant’s fingers pointed in the same direction. The left-right movement of the respiratory band and the up-down movement of the mannequin hand were asynchronous due to non-movement of the mannequin hand.

SUPPLEMENTARY VIDEO S5 Representative video in the conditions of spatial incongruence and temporal synchronization. The mannequin’s fingertips were pointing toward the participant and the palm was pointing upward. The left-right movement of the respiratory band and the up-down movement of the mannequin hand were synchronized.

SUPPLEMENTARY VIDEO S6 Representative video in the conditions of spatial incongruence and temporal asynchronization. The mannequin’s fingertips were pointing toward the participant and the palm was pointing upward. The left-right movement of the respiratory band and the up-down movement of the mannequin hand were asynchronous due to random movement of mannequin hand.

SUPPLEMENTARY VIDEO S7 Representative video in the conditions of spatial incongruence and temporal constant speed. The mannequin’s fingertips were pointing toward the participant and the palm was pointing upward. The left-right movement of the respiratory band and the up-down movement of the mannequin hand were asynchronous due to constant movement of the mannequin hand.

SUPPLEMENTARY VIDEO S8 Representative video in the conditions of spatial incongruence and temporal static. The mannequin’s fingertips were pointing toward the participant and the palm was pointing upward. The left-right movement of the respiratory band and the up-down movement of the mannequin hand were asynchronous due to non-movement of the mannequin hand.
